# Replacing what’s lost: a new era of stem cell therapy for Parkinson’s disease

**DOI:** 10.1186/s40035-019-0180-x

**Published:** 2020-01-07

**Authors:** Yong Fan, Winanto, Shi-Yan Ng

**Affiliations:** 1 The Third Affiliated Hospital of Guangzhou Medical University, Guangzhou, 510150 China; 2 Institute of Molecular and Cell Biology, A*STAR Research Entities, Singapore, 138673 Singapore; 3 Yong Loo Lin School of Medicine (Physiology), National University of Singapore, Singapore, 117456 Singapore; 4 National Neuroscience Institute, Singapore, 308433 Singapore

**Keywords:** Parkinson’s disease, Induced pluripotent stem cells, Cell therapy, Regenerative medicine

## Abstract

**Background:**

Stem cells hold tremendous promise for regenerative medicine because they can be expanded infinitely, giving rise to large numbers of differentiated cells required for transplantation. Stem cells can be derived from fetal sources, embryonic origins (embryonic stem cells or ESCs) or reprogrammed from adult cell types (induced pluripotent stem cells or iPSCs). One unique property of stem cells is their ability to be directed towards specific cell types of clinical interest, and can mature into functional cell types in vivo. While transplantations of fetal or ESC-derived tissues are known to illicit a host immunogenic response, autologous transplantations using cell types derived from one’s own iPSCs eliminate risks of tissue rejection and reduce the need for immunosuppressants. However, even with these benefits, cell therapy comes with significant hurdles that researchers are starting to overcome. In this review, we will discuss the various steps to ensure safety, efficacy and clinical practicality of cell replacement therapy in neurodegenerative diseases, in particular, Parkinson’s disease.

**Main body:**

Parkinson’s disease (PD) results from a loss of dopaminergic neurons from the substantia nigra and is an ideal target for cell replacement therapy. Early trials using fetal midbrain material in the late 1980s have resulted in long term benefit for some patients, but there were multiple shortcomings including the non-standardization and quality control of the transplanted fetal material, and graft-induced dyskinesia that some patients experience as a result. On the other hand, pluripotent stem cells such as ESCs and iPSCs serve as an attractive source of cells because they can be indefinitely cultured and is an unlimited source of cells. Stem cell technologies and our understanding of the developmental potential of ESCs and iPSCs have deepened in recent years and a clinical trial for iPSC-derived dopaminergic cells is currently undergoing for PD patients in Japan. In this focused review, we will first provide a historical aspect of cell therapies in PD, and then discuss the various challenges pertaining to the safety and efficacy of stem cell-based cell transplantations, and how these hurdles were eventually overcome.

**Conclusion:**

With the maturity of the iPSC technology, cell transplantation appears to be a safe and effective therapy. Grafts in non-human primates survive and remain functional for more than 2 years after transplantation, with no signs of tumorigenesis, indicating safety and efficacy of the treatment. However, immunosuppressants are still required because of the lack of “universal stem cells” that would not evoke an immune response. The results of ongoing and upcoming trials by a global consortium known as GForce-PD would be highly anticipated because the success of these trials would open up possibilities for using cell therapy for the treatment of PD and other degenerative diseases.

## Background

The discovery of embryonic stem cells (ESCs) and their ability to both self-renew, enabling unlimited expansions of these naïve cells, and their pluripotent properties that allow the derivation of any adult differentiated cell types, fuelled the hopes of patients, researchers and clinicians that cell transplantation as a form of therapy would cure debilitating neurodegenerative disorders where neurons are lost. The subsequent invention of human induced pluripotent stem cells (iPSCs) by Yamanaka and colleagues [53] further added on to the hype because of the belief that transplantation of one’s own stem cell-derived products (known as autologous transplantation) would evade the body’s innate immune surveillance. Immunosuppressant drugs can be entirely avoided, and success rates would be improved.

More than a decade after the discovery of iPSCs, we still do not have a stem cell therapy, but the first clinical trials involving human ESC- and iPSC-derived products have started to take place and a therapy may soon become available. This long and arduous journey reflects the vast obstacles that stem cell scientists have just begun to overcome. In this review article, we aim to highlight and discuss a number of hurdles in using stem cell-derived products for cell replacement therapy, their solutions, and what are our realistic expectations of them in this new era of stem cell therapy, focusing on Parkinson’s disease (PD).

## Main text

### PD as a candidate for stem cell therapy

Neurodegeneration broadly involves the progressive loss of neurons in the nervous system. Recent evidence show that neurons begin losing their normal functions and morphologies even before neuronal death, suggesting that simply preventing these neurons from dying is unlikely to be an effective therapeutic approach [50]. As such, unless there are therapeutic strategies that preserve the structure and function of neurons, cell transplantation still appears to be the most effective approach. However, without a deep understanding of the biology of the diseases and pathological mechanisms, cell replacement therapy is still very much an empirical “trial-and-error” approach. For instance, cell transplantation is likely to be more successful for PD than for a neurodegegenrative disease that simultaneously affects multiple regions in the brain. In PD, dopaminergic neurons in a specific anatomical region, known as the substantia nigra pars compacta (SNpc), are lost. Along the nigrostriatal pathway, SNpc dopaminergic neurons innervate the dorsal striatum where they release the neurotransmitter dopamine. Loss of dopaminergic neurons in the SNpc is one of the main pathological feature in PD, and is responsible for the symptomatic motor deficits of PD.

### A historical perspective of fetal cell transplantations in Parkinson’s disease

Grafting of fetal midbrain tissues has been considered a therapeutic approach for PD since the 1980s [5]. The first double blind study comparing transplantations of fetal ventral midbrain tissues versus a sham surgery control was performed in 2001, where patients who received bilateral injections of fetal cells into the putamen demonstrated a modest recovery compared to the sham group [18]. There was no significant improvements in older patients more than 60 years old, and in patients who received the fetal grafts, some eventually developed dyskinesias and dystonia [18, 41]. These unpredictable and undesirable side effects from fetal grafts, along with the introduction of deep brain stimulation in 1997 to treat tremors in PD, cell transplantations for PD were temporarily halted until recently when the TRANSEURO consortium revisited fetal ventral midbrain grafts for PD treatment in a multi-center trial (NCT01898390) [2]. Results of these early fetal cell transplantation studies tended to be highly variable because of the lack of standardization in fetal material to be transplanted. However, key conclusions could be drawn from these studies. A number of retrospective studies examined post-mortem brains of PD patients who survived more than 10 years after receiving fetal midbrain transplants in the early 1990s [20, 32, 34]. These concluded that grafted dopaminergic neurons remain healthy and functional, resulting in long term symptomatic relief in some of the patients in the study. Even though a small percentage of grafted neurons developed Lewy bodies that may suggest host-to-graft disease propagation, majority of the neurons were healthy and functional for more than a decade in these patients. In short, transplantation of fetal ventral midbrain tissue into the striatum of PD patients has provided a proof-of-principle that cell therapies can provide long-term clinical benefits.

### Cell replacement therapy for Parkinson’s disease – hurdles and solutions

A successful cell transplantation has to be safe, well-tolerated by the recipient, and efficacious in reversing the symptoms of disease. It has been extremely well-established that loss of dopamine in the striatum is responsible for PD. Levodopa, which is a dopamine precursor, has been used to treat PD patients since that replaces the dopamine that is lost [35, 39]. Moreover, numerous animal studies have shown that transplanting dopaminergic neurons improves motor symptoms in PD animals [19, 25, 29, 43]. Even though there was sufficient evidence supporting that dopaminergic cells are clinically relevant for cell therapy in PD, there were aspects relating to their safety and reproducibility. Below, we list some of the hurdles that were eventually overcome so that stem cell derivatives can be used in the clinic.

#### Hurdle 1 – deriving the right neural cell type for transplantation

Dopaminergic neurons were first derived from human ESCs with the help of mouse stromal feeder cells such as MS5 or PA6 [24, 43], which is neither chemically-defined nor rigorously reproducible. Although it has been demonstrated that Wnt and Sonic hedgehog secreted by MS5 or PA6 were responsible for the dopaminergic induction properties [43, 52], results from different labs show lack of reproducibility using this non-chemically defined method [56, 57]. Although these initial reports of human ESC-derived dopaminergic neurons express markers of SNpc dopaminergic neuron fate (such as LMX1, EN-1 and NURR1), these cultures were also found to be contaminated with other neural types, including GABAergic, cholinergic and serotonergic neurons that are not relevant in PD [24].

As researchers understood better the signalling pathways that are involved in specific derivation of SNpc dopaminergic neurons [1, 7, 8, 22, 58], differentiation protocols also improved. Following the rules of developmental biology, Studer and colleagues found that the simultaneous inhibition of BMP and SMAD pathways using recombinant Noggin and SB431542 reproducibly generated more than 80% of PAX6 and SOX1-expressing neuroectoderm progenitors [9]. During development, Sonic Hedgehog (SHH) secreted by the floorplate and Fibroblast Growth Factor 8 (FGF8) secreted by the isthmus are thought to be key molecules involved in the specification and differentiation of SNpc dopaminergic neurons [48]. Recent protocols, however, also implicate the roles of Wnt signalling in floorplate specification. Using CHIR99021, a GSK3B inhibitor that acts as a Wnt agonist, Parmar and colleagues determined that lack of Wnt signalling during neural induction results in a rostral fate while a high level of Wnt activation led to a caudal (spinal cord) fate. Intermediate levels of Wnt signalling induced by 0.7 μM CHIR99021 was optimal for midbrain and floorplate specification [27]. Based on this paper that describes a chemically-defined protocol, many labs have since reproducibly generated LMX1^+^FOXA2^+^ floorplate progenitors and subsequent dopaminergic neurons using multiple human ESC and iPSC lines [25, 42, 46], reflecting the robustness of the protocol. However, more recently, [28] performed deep sequencing of more than 30 human ESC-derived midbrain tissues and correlated this data with graft outcome, and found that floorplate markers such as LMX1A, FOXA2 and CORIN do not correlate with better graft outcome. Rather, by introducing FGF8b into floorplate differentiation cultures in a temporal manner, Kirkeby et al. demonstrated that cultures that acquired a caudal midbrain fate (EN1^+^CNPY1^+^BARHL1^low^) promotes high dopaminergic graft volume, density and yield. On the contrary, floorplate cultures that were not exposed to FGF8b represents rostral midbrain cell types that had poor dopaminergic yield after transplantation [28]. This indicates that derivation of caudal midbrain dopaminergic cultures is important for cell transplantation.

#### Hurdle 2 – eliminating the risk of tumorigenesis

Pluripotent stem cells that are refractory to differentiation cues or remain dormant in cultures can be hazardous when transplanted into patients due to the risk of tumorigenesis. Pluripotent stem cells form teratomas when transplanted into rodents [33], and transplantation of unpurified cultures into rodent brains also result in the formation of tumors. Multiple methods have been proposed to limit this risk, broadly categorized into elimination of stem cells from the final cultures and purification of the desired cell types. For instance, undifferentiated pluripotent stem cells express unique cell surface markers such as SSEA-3, SSEA-4, TRA-1-60 and TRA-1-81 [6]. Though this “negative-sorting” strategy has been shown to work in eliminating undifferentiated pluripotent stem cells in a mixed culture [17], this still does not enrich for the desired cell types that will repopulate the graft.

Another strategy to eliminate tumor formation is the use pluripotent stem cells with limited proliferation capacity. With the idea that stem cells with critically-shortened telomeres would trigger apoptosis and therefore rid the eventual cultures of tumorigenic stem cells, Liu and colleagues generated telomerase knockout (TERT^−/−^) human ESCs and showed that telomerase inactivation resulted in progressive shortening of telomeres in ESCs and their derived cell types. To test for tumorigenicity, TERT^−/−^ ESCs were injected into the midbrains of immunodeficient mice, where they were found to be incapable of tumor formation 4 weeks after injection. In contrast, injection of TERT^+/+^ ESCs resulted in large tumor masses [33]. While using stem cells with limited proliferation appears to be a feasible idea, specific issues relating to its eventual efficacy have to be raised. There is evidence that neural stem cells with shortened telomeres are less effective in neuronal differentiation, and poorer neurite outgrowth [16]. It is also now known that telomerase deficiency induces cellular senescence and a premature aging phenotype. TERT-knockout mice have short dysfunctional telomeres, smaller brain sizes and sustained DNA damage signaling [23]. With these aging phenotypes accumulating in TERT^−/−^ tissues, it would appear counter-productive to graft “aging cells” to treat a neurodegenerative disease.

There are also multiple efforts aimed at elucidating the cell surface proteome of midbrain dopaminergic progenitors and neurons, with the idea that a cocktail of cell surface antibodies can be used to purify these desired neural types for transplantation. In this regard, multiple labs have succeeded in profiling the surface proteome of floorplate progenitor cells using a variety of methods. The Kim group was the first to use Corin, a transmembrane serine protease, as a cell surface marker of floorplate progenitors, along with an Otx2 genetic reporter [11]. Corin is known to be expressed in the floorplate in the developing nervous system while Otx2 is a transcription factor that can induce the midbrain phenotypes when ectopically expressed [13]. Using this strategy, they purified Otx2^+^Corin^+^ cells from differentiating mouse ESCs, and found that these sorted cells differentiate efficiently into midbrain dopaminergic neurons with almost 80% efficiency. Transplantation of the Otx2^+^Corin^+^ cells into the striatum of mice with PD phenotypes showed an improvement in behaviour that is attributed to successful engraftment and function of the cells in vivo. Notably, graft volume using sorted cells was significantly smaller compared to the use of unsorted cells presumably due to lack of teratoma formation. Dopaminergic density and total number of dopaminergic neurons were also significantly higher than unsorted controls, indicative of in vivo differentiation of the Otx2^+^Corin^+^ floorplate progenitors towards functional midbrain dopaminergic neurons. Subsequently, the labs of Takahashi and Parmar showed that the use of CORIN alone as a single sorting marker for human pluripotent stem cell-derived floorplate progenitors was sufficient to prevent tumor formation while maintaining efficient differentiation into functional dopaminergic neurons after transplantation [14]. They also found that CD166 (ALCAM) tends to be co-expressed with CORIN in the floorplate progenitors. Knöbel and colleagues subsequently discovered that CD47 (also known as integrin-associated protein or IAP) also reproducibly marks the floor plate progenitors [31]. Using a genome-wide method, Rubin and colleagues purified FOXA2^+^LMX1^+^ floorplate progenitors and analysed the transcriptome profile for mRNAs encoding surface and transmembrane proteins [42] and independently confirmed that CORIN, CD166 and CD47 are enriched in the floorplate progenitors, while CXCR4 is depleted. This CORIN^+^CD166^+^CXCR4^−^ “triple sorting” strategy appears to be more efficient in generating dopaminergic neurons than the use of single CORIN-only sorting.

Other groups have also investigated alternative markers for purification of dopaminergic progenitors differentiated from ESCs and iPSCs. With the view that CORIN alone is not stringent enough as a floorplate marker, Takahashi’s lab isolated CORIN^+^LMX1A:GFP^+^ progenitors for transcriptional profiling. They subsequently found that LRTM1 is a transmembrane protein that is specific for CORIN^+^LMX1A^+^ floorplate progenitors. While LRTM1^+^ floorplate progenitors also differentiate into dopaminergic neurons in a primate PD model, and resulted in motor improvement with no teratoma formation [49], it is unclear how this LRTM1 sorting strategy compares with CORIN sorting for transplantation in PD. Separately, using a proteomics-based approach, Fathi et al., showed that LMX1A^+^ dopaminergic progenitors express both PSA-NCAM and Contactin-2 (CNTN2) on their surface and that transplantation of CNTN2^+^ progenitors enhanced dopamine release in the host brain compared to unsorted cells, along with associated improvements in PD symptoms in rodents [15]. Likewise, it is not clear how CNTN2 sorting compares to CORIN sorting.

Several lines of evidence suggest that transplanting a heterogeneous cell population is potentially hazardous, increasing the risk of tumorigenesis due to presence of undifferentiated stem cells [11, 14]. To overcome this, a number of surface markers specific to floorplate progenitors have been identified, including CORIN, CD166, CD47, LRTM1 and CNTN2. However, only the use of CORIN sorting has been extensively characterized to date [14, 25, 38, 42]. Even though purification of CORIN-expressing floorplate progenitors was effective in eliminating tumorigenic stem cells, a recent report by Parmar’s lab suggest that CORIN is not a good predictive marker for dopaminergic differentiation and yield [28]. Therefore, it is critical that the other surface markers and combinations of markers are evaluated vis-à-vis a single CORIN sort.

#### Hurdle 3 – transplanting floorplate progenitor cells, fully differentiated SNpc dopaminergic neurons or an intermediate?

PD is caused by loss of dopaminergic neurons in the SNpc and intuitively, it is logical to transplant fully differentiated SNpc dopaminergic neurons to replace those that are lost. Several groups have shown that this is possible: Kriks, Studer and colleagues have shown that floorplate-derived dopaminergic neurons differentiated from human ESCs engraft efficiently in mouse, rat and monkey brains [29]. Isacson and colleagues also transplanted NCAM^+^CD29^low^ DA neurons into rats and non-human primates that demonstrated restoration of motor function in 6-hydroxydopamine (6-OHDA)-lesioned rats, as well as survival, integration and function of the grafts in primate brains up to 2 years after autologous transplantation without immunosuppression [21, 51]. However, Chung and colleagues have shown that transplantation of Corin^+^Otx2^+^ floorplate progenitors into 6-OHDA-treated mice were also capable of differentiation into TH^+^ dopaminergic neurons in vivo and ameliorated the behavioral deficits in these mice [11]. Therefore, this begs the question as to the ideal cell stage for transplantation: newly formed floorplate progenitors, restricted dopaminergic progenitors or fully differentiated dopaminergic neurons? To answer this problem, Zeng and colleagues transplanted progenitors and neurons at various stages of the dopaminergic differentiation protocol into mice. Starting with human ESCs, they followed the dopaminergic differentiation protocol from [27] and transplanted cells derived from three stages of differentiation into mice with 6-OHDA-lesioned brains, namely: day 16 OTX2^+^LMX1A^+^ floorplate progenitors, day 25 NURR1^+^ dopaminergic progenitors or day 35 TH^+^MAP 2^+^ dopaminergic neurons. Analyses of mice brains 3 months post-transplantation concluded that graft sizes using day 35 cells were the smallest, while number of neurons were fewest in the brains grafted with day 16 cells. Grafts using day 25 cells had the highest density of dopaminergic neurons. While transplantation of day 16 cells did not reduce the amphetamine-induced rotations in these PD mice, the use of day 25 and day 35 cells performed equally well in reducing this behavioural deficit [46].

The groups of Malin Parmar and Jun Takahashi have made significant progress in defining the “ideal” cell state for PD transplantations using human pluripotent stem cell-derived products. These studies have demonstrated that human pluripotent stem cells could be differentiated towards floorplate progenitors within 12 days, where they were sorted for CORIN, and further cultured until day 28. These dopaminergic precursors at day 28 have been transplanted into rat and monkey models of PD, where the graft survived and matured in vivo into dopaminergic neurons. Serotonin neurons were absent in these grafts. As a result, the lesioned animals also demonstrated significant motor recovery without dyskinesia or tumor formation [14, 25]. In a slight variation of this protocol, Parmar, Kirkeby and co-workers also report a 16-day protocol to obtain ventral midbrain dopaminergic precursors with high purity that is compatible with intracerebral transplantations [38]. Therefore, it is widely accepted that “dopaminergic precursors” beyond the floorplate progenitor stage but before formation of TH^+^ dopaminergic neurons are most efficacious for eventual graft survival, integration and function.

#### Hurdle 4 – source of stem cells: autologous or allogeneic? Fetal, ESC or iPSC-derived?

The initial source of stem cell is an important question to tackle to evade immunogenic response, especially if long-term use of immunosuppressants should be avoided. The use of autologous stem cells (stem cells derived from one’s own body) would be perfect for purposes of transplantation since the risk of tissue rejection would be extremely low. This has been verified by the Isacson group, where they made iPSCs from cynomolgus monkeys and transplanted the iPSC-derived dopaminergic neurons back to the same monkey. They reported survival of the graft for up to 2 years post-transplantation and improvements to motor function and activity with no administration of immunosuppressant drugs [21], confirming the long-term function and efficacy of autologous grafts. However, one major disadvantage of autologous grafts is that transplanted cells would still retain the genetic mutations or risk factors that contributed to PD in the first place. Moreover, it would be an extremely costly effort, both in terms of resources and time, to generate iPSCs from each patient and differentiate them into suitable cells for transplantation. Given that there is huge amount of variability between iPSCs derived from different individuals, it is also not straightforward to perform quality control checks on each batch of cells derived from different patients. Therefore, while autologous stem cell transplants appear to be a good solution, many practical and implementation issues prevent that from being the most ideal source of stem cells for transplants. Likewise, the same argument can be applied to fetal tissues, which is both scarce and fundamentally variable since the stage of development and the genetic makeup of each fetal material can be vastly different. Another drawback of fetal midbrain tissue transplantation was the development of graft-induced dyskinesia in some patients, which was partly attributed to the presence of large numbers of serotonin neurons in the fetal midbrain tissue [45].

Increasingly, single cell analyses have been used to assess composition and quality of stem cell-derived tissues, vis-à-vis fetal tissues. ESC- and iPSC-derived dopaminergic cultures using Krik’s differentiation protocol [29] yielded multiple cell types that were found in the human fetal midbrain, suggesting that pluripotent stem cell-derived floorplate cells recapitulated key stages in ventral midbrain development [30]. A recent study also interrogated grafts derived from human pluripotent stem cells versus grafts derived from fetal ventral midbrain at the single cell resolution. Their findings suggest that ESC and iPSC-derived grafts were ideal for transplantation for the following reasons: 1) fewer astrocytes were derived from grafts originating from ESC and iPSC; 2) serotonin neurons were completely undetected by single cell RNA-seq, confirming the safety of these grafts; 3) vascular leptomeningeal cells were detected in grafts derived from ESCs and iPSCs but not in fetal midbrain-derived grafts. It is suggested that these brain vascular cells could promote graft survival and maturation by promoting rapid re-vascularization and re-establishment of the blood-brain-barrier [55].

Since personalized stem cell therapy poses a number of issues with regards to reproducibility and resources, allogenic transplants using either matched ESCs or iPSCs appear to be most ideal. The feasibility of banking, differentiation, and eventual quality control of a smaller number of routinely used cell lines would possibly make cell therapy accessible to most patients. Human leukocyte antigen (HLA) matching can be performed to ensure that risk of tissue rejection post-implantation is reduced. It has been proposed that generation of iPSC banks for HLA-matched tissue transplantations can fulfill the cell therapy needs in some populations with a minimal requirement for immunosuppression [54]. For instance, ([36, 40] estimated that between 50 to 140 donor iPSC lines would be required to provide HLA-matched tissues for 90% of the Japanese population. For the rest of the world, umbilical cord lining cells, which have been shown to be immunologically naïve, may be ideal as a cell source. Umbilical cord lining-derived epithelial cells (CLECs) transplanted into immunocompetent mice were capable of long-term maintenance without the need for immunosuppressants [59]. These CLECs have been converted into iPSCs termed cord lining induced pluripotent stem cells or CLiPS that can differentiate into functional midbrain dopaminergic neurons that does not appear to illicit an immune response in host animals after transplant. Transplanted DA neurons derived from CLiPS alleviate the PD symptoms in a immuno-competent mice lesioned with 6-OHDA without the need for immunosuppressants (personal correspondence with Prof Lim Kah Leong and CellResearch Corporation). Current work is ongoing to understand the mechanisms contributing to the immune-privileged properties of the CLiPS.

Recently, the idea of “universal stem cells” has also been gaining traction and is primed for cell transplantation studies in regenerative medicine. An important milestone that was achieved this year (2019) by Schrepfer and colleagues was to use CRISPR/Cas9-mediated genome editing strategies to delete major histocompatibility complex (MHC) Class I and II genes and the simultaneous overexpression of “don’t-eat-me” signal CD47 to evade natural killer (NK) cells [12]. These “hypoimmunogenic” iPSCs were subsequently differentiated into cardiovascular cell types that grafted long-term in MHC-mismatched rodents without the use of immunosuppression. The authors also noted that CD47 overexpression was critical in the complete evasion of immune rejection by NK cells, and the immediate concern would be the safety of these cells for transplantation since CD47 is known to be overexpressed in multiple cancers [10], and more studies have to be done on these universal stem cells to demonstrate the lack of tumorigenesis in the long term.

#### Hurdle 5 – how many cells to transplant? Where to transplant?

In PD, the SNpc dopaminergic neurons are selectively lost. These neurons project to the putamen and caudate nuclei in the dorsal striatum where they release dopamine to the recipient cells. As such, many SNpc replacement therapy trials in animals graft dopaminergic neurons ectopically into the striatum of recipients [19, 25]. However, in a study where Parmar and colleagues sought to investigate the capacity for long-distance and target innervation of ESC-derived dopaminergic neurons grafted into the SNpc, they found that these grafted neurons extended long projections that were more than 10 mm along the medial forebrain bundle and nigrostriatal pathway, and densely innervated the caudate-putamen, amongst other brain structures [19]. Behavioral studies were however, not performed in this study and it was therefore not possible to access if grafting of neurons in the SNpc has therapeutic effects in the PD animals. However, it is noteworthy that the human brain is much larger than rodent and macaque brains, and the length of neuronal projections may still not be enough to innervate the striatum if the neurons were to be grafted in the SNpc. Therefore, grafting into the putamen, where SNpc dopaminergic neurons eventually innervate, remains the most realistic approach.

Multiple studies have now established that stem cell-derived dopaminergic grafts survive in host animals survive for at least 2 years after transplantation, while fetal-derived dopaminergic grafts in human recipients remain viable even after more than a decade. Observations from these various studies point out that a single dose of transplantation was sufficient for prolonged survival and function of the graft. In studies using grafts of human midbrain material, Rath et al. established that as few as 650 surviving TH^+^ dopaminergic neurons was sufficient for behavioural and motor improvements in a rat PD model [47]. Using these as guidelines for efficacy, the Takahashi group used 4.8 million cells per macaque, split into 6 tracts of 4 injections per tract per side, with each injection containing 100,000 cells [26]. 6 months post-transplantation, a maximum of 126,000 TH^+^ dopaminergic neurons remained surviving where iPSC-derived cultures at day 42 were grafted. They also found that even in the macaque with only 16,000 TH^+^ cells (the lowest recorded in their study), the PD score improvement and motor function was equivalent to the animal with more surviving TH^+^ cells, suggesting that a minimum of 16,000 TH^+^ cells is sufficient to produce a saturating effect [25].

### Clinical trials for Parkinson’s disease and what should we expect?

While transplantations of fetal midbrain material continue to be explored under the TRANSEURO trial, the use of fetal tissues is associated with several ethical and standardization issues that may not be a realistic cell therapy option for PD, especially since cells from approximately 4 fetuses would be required for a single patient [3, 18]. A global consortium, GForce-PD, has also been set up, bringing together major research teams in Europe, USA and Japan to work on developing stem cell-derived neural cell therapies for PD [4]. The EUROPEAN STEM-PD, NYSTEM-PD, CiRA Trial and Summit for PD Trial are four of such planned clinical trials by members of the GForce-PD using either human ESCs (EUROPEAN STEM-PD and NYSTEM-PD) or iPSCs (CiRA and Summit for PD) as initial cell source [3]. On 1 August 2018, a cell transplantation trial for PD led by Jun Takahashi, Ryosuke Takahashi and colleagues in Kyoto University’s Center for iPS Cell Research and Application (CiRA) had begun to recruit their first patients. The key features and considerations of these upcoming and ongoing trials are previously reviewed in [3].

One of the biggest hurdles of pluripotent stem cell therapies is that of large scale production of homogeneous cell types in a reproducible manner that also conforms to current Good Manufacturing Practices (cGMP) standards. Clinical grade ESCs and iPSCs will have to be stringently evaluated for tumorigenicity, genomic stability and other safety aspects, will be cultured and differentiated into dopaminergic progenitors in a standardized cGMP-compliant facility. Since pluripotent stem cells and their neural derivatives require specific coatings of extracellular matrix proteins on culture vessels, matrices such as Matrigel or mouse laminin are not suitable because of their xenogenic components. To overcome this, multiple groups have used xeno-free laminin fragments such as laminin-111, laminin-421 [28], laminin-511 [14] and laminin-521 [37, 38] for large-scale culture and differentiation of ESCs and iPSCs. As part of the cell manufacturing process to ensure homogeneity of cells, the CiRA trial implements CORIN sorting to purify the floorplate progenitor cells that will eventually give rise to dopaminergic neurons. In non-human primate studies where CORIN-purified midbrain progenitors were transplanted into lesioned animals, the grafted putamen had extensive innervation by TH^+^ fibers. Furthermore, implanting CORIN^+^ cells dramatically reduces the tumorigenic risk, and eliminates serotonergic neurons that results in dyskinesia [14, 25]. Therefore, even though CORIN appears to be a poor predictive marker of transplantation outcome as it is strongly associated with a rostral midbrain fate [28], the current preclinical evidence suggests that the benefits of CORIN sorting far outweighs the potential disadvantages.

**Fig. 1:**
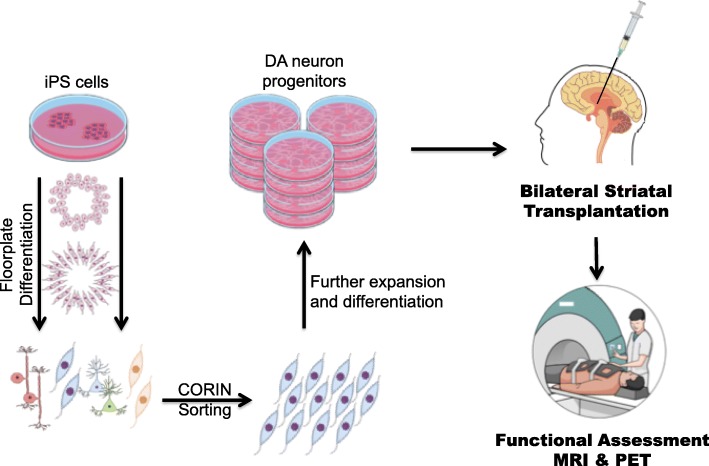
Schematic of workflow for derivation of therapeutic-grade dopaminergic precursors. Undifferentiated iPSCs are propagated and differentiated on cGMP-grade laminin-511 fragments that are xeno-free. Sorting for CORIN-expressing cells ensures a highly-enriched population of floorplate progenitors are used for subsequent expansion and differentiation. Large numbers of purified dopaminergic precursors would be obtained, providing enough material for transplantation of 4.8 million cells per patient. Each patient from the CiRA clinical trial would be observed for at least 2 years after transplantation

In the ongoing CiRA Phase I/II trial, successful preclinical research performed previously by Takahashi and team will serve as the guiding fundamentals for the cell transplantation in patients (Fig. 1). Seven mid-stage patients who has PD for more than 5 years were recruited. The rationale behind treating mid-stage, rather than severe end-stage PD patients was that in the latter patients, the striatal and innervating cortical neurons would have degenerated to the point that they were no longer responsive to dopamine secreted by the transplanted neurons. Moreover, previous studies of fetal cell transplantations showed that it has little benefit for patients suffering from severe PD [44]. As such, these seven recruited patients should also have L-dopa response of more than 30% [3]. In this trial, iPSCs from an HLA-homozygous donor would be used, which is estimated to be compatible with approximately 17% of the Japanese population. Each patient will receive 4.8 million human iPSC-derived CORIN+ dopaminergic progenitors implanted into their bilateral putamen via stereotaxic brain surgery. However, since this would be an allogenic transplantation, patients will receive tacrolimus (also known as FK506), a standard immunosuppressant, to prevent possible graft rejection.

Patients will be observed for at least 2 years post-transplantation, with qualitative and quantitative motor, cognitive, psychiatric and quality of life assessments, as well as imaging tests to monitor the graft. Specifically, magnetic resonance imaging (MRI) would be used to monitor graft survival and proliferation by measuring the volume of grafts, which are recognized as hyperintensity areas [25]. Secondly, positron emission tomography (PET) using [^18^F] fluorothymidine can detect actively proliferating cells within the graft, which could signal tumorigenesis. [^18^F]DOPA-PET would also be used to monitor the dopamine content of the graft and can be used to assess graft function. A successful trial would be defined as one with no adverse reactions, i.e. tumor formation resulting from the grafts, and shows significant improvements to motor and non-motor assessments.

## Conclusion

Preclinical studies have demonstrated the vast potential of cell replacement therapy for treating neurodegenerative diseases such as PD. Protocols for derivation of dopaminergic precursors are well established and characterized, giving rise to large numbers of clinically-relevant cells. The CORIN sorting strategy is a key step in the derivation protocol and serves not just to eliminate tumorigenesis, but also to remove serotonin neurons and precursors which are known to result in dyskinesia when transplanted into patients. The results of the Phase I/II trial performed by Kyoto University’s CiRA would be important because this represents a first-in-human trial using neural cells derived from pluripotent stem cells. In addition, other members of the GForce-PD consortium are each planning for their respective trials to take place in the next few years and the results of these trials would be highly anticipated because the success of the trial would open up possibilities for using cell therapy for the treatment of other degenerative diseases.

## Data Availability

Not applicable.

## Abbreviations

6-OHDA: 6-hydroxydopamine
cGMP: Current Good Manufacturing Practices
CiRA: Center for iPS Cell Research and Application
CNTN2: Contactin-2
DAT: Dopamine transporter
ESCs: Embryonic stem cells
FGF8: Fibroblast Growth Factor 8
HLA: Human leukocyte antigen
iPSCs: Induced pluripotent stem cells
NK: Natural killer
PD: Parkinson’s disease
PET: Positron emission tomography
SHH: Sonic Hedgehog
SNpc: Substantia nigra par compacta
TERT: Telomerase reverse transcriptase
